# Dissecting the causal role of immunophenotypes in primary sclerosing cholangitis risk: A Mendelian randomization study

**DOI:** 10.1097/MD.0000000000038626

**Published:** 2024-06-28

**Authors:** Jie Zhou, Haitao Wang, Yixin Xu, Zhilin Liu

**Affiliations:** a Department of Gastrointestinal Surgery, the Third Affiliated Hospital of Soochow University, Changzhou, China; b Department of General Surgery, Wujin Hospital Affiliated with Jiangsu University, Changzhou, China

**Keywords:** causal relationship, hepatobiliary system, immunity, single nucleotide polymorphism

## Abstract

Primary sclerosing cholangitis (PSC), a chronic cholestatic liver condition, is frequently associated with inflammatory bowel disease. Specific immune cells have been implicated in PSC pathogenesis with the emergence of the “microbiota” and “gut lymphocyte homing” hypotheses, albeit their identities remain controversial. The first genome-wide association analysis leveraged nonoverlapping data from 3757 Europeans to evaluate 731 immunophenotypes. A genome-wide association analysis comprising 2871 cases and 12,019 controls yielded summary statistics for PSC. An inverse-variance weighted (IVW) analysis was performed to identify immunophenotypes causally related to PSC, and the results were validated using weighted mode, MR-Egger, and weighted median methods. Comprehensive sensitivity analyses were performed to verify the robustness, heterogeneity, and horizontal pleiotropy of the results. IVW analysis revealed 26 immune traits exhibiting causal associations with PSC. CD3 on HLA-DR+ CD4+ (IVW odds ratio [OR]: 0.904; 95% confidence interval [CI]: 0.828–0.986, *P = *.023) and CD3 on secreting Treg (IVW OR: 0.893; 95% CI: 0.823–0.969, *P = *.007) were negatively associated with PSC susceptibility and demonstrated high consistency across the 3 validation methods. Moreover, 7 other immune traits, including CD39+ resting Treg absolute cell (IVW OR = 1.083, 95% CI: 1.013–1.157, *P = *.019), CD39+ secreting Treg absolute cell (IVW OR = 1.063, 95% CI: 1.012–1.118, *P = *.015), CD3 on naive CD8br (IVW OR = 0.907, 95% CI: 0.835–0.986, *P = *.022), CD3 on CD39+ activated Treg (IVW OR = 0.927, 95% CI: 0.864–0.994, *P = *.034), CD28 on resting Treg (IVW OR = 0.724, 95% CI: 0.630–0.833, *P = *5.95E-06), and CD39 on CD39+ CD4+ (IVW OR = 1.055, 95% CI: 1.001–1.112, *P = *.044) exhibited consistent results in the Weighted Median and Weighted Mode validation methods. Moreover, no significant heterogeneity or horizontal pleiotropy was observed across the single nucleotide polymorphisms. The leave-one-out results revealed that sequentially eliminating each single nucleotide polymorphism had no significant influence on model effect estimates or qualitative inference. This study evaluated potential causal links between 731 immune traits and PSC susceptibility. Twenty-six immune traits were identified using the IVW method. Verification across multiple methods revealed 9 immune traits with a plausible causal connection to PSC. These findings may uncover mechanistic pathways and novel therapeutic approaches.

## 1. Introduction

Primary sclerosing cholangitis (PSC) is a chronic cholestatic liver disease characterized by bile duct damage inside and/or outside the liver.^[1]^ Although the incidence of PSC varies geographically, it affects up to 1.3 per 100,000 persons in Northern Europe each year and is on the rise.^[2]^ A population-based Dutch study discovered a median survival of 21.3 years from diagnosis to liver transplant or disease-related death. Besides, the median waitlist survival on the United Network for Organ Sharing was 13.2 years.^[3]^

Furthermore, while several mechanistic theories have been proposed, and genetic, immunologic, and environmental factors appear to play a role in the development of PSC, the precise pathogenesis of the disease is unknown.^[2,4]^ Concurrently, inflammatory bowel diseases (IBDs), which affect 70% of PSC patients, are the most potent recognized clinical risk factor.^[5,6]^ The “microbiota hypothesis” now proposes that dysregulated intestinal microbes and their molecules enter the liver via the portal vein and induce aberrant cholangiocyte responses, ultimately contributing to PSC pathogenesis.^[2,7]^ The close link between PSC and IBDs also suggests another theory for PSC pathogenesis–the “gut lymphocyte homing” hypothesis. It is hypothesized that intestinal T cells become activated inside the intestine-associated lymphoid tissue and then are recruited to the liver due to aberrant expression of adhesion molecules on periportal endothelial cells. These activated intestinal T cells can initiate immune-mediated damage to the liver.^[7,8]^ Moreover, researchers have hypothesized that cholangiocytes may actively participate in the pathogenesis of PSC by secreting proinflammatory cytokines, mediating recruitment, and stimulation of T cells, or developing a senescent phenotype.^[4]^ However, the degree to which the aforementioned T cell subsets and other immune cells, including natural killer cells, B cells, and natural killer T cells, contribute to PSC pathogenesis is debatable.^[1,4]^ Of note, a lack of understanding of PSC pathogenesis precludes the development of effective therapeutics.

Mendelian randomization (MR) examines the link between a proxy based on genetic variants associated with exposure and outcomes and infers potential causal associations using the genetic variants as exposure instrumental variables (IVs).^[9,10]^ In this view, MR is a valuable approach for elucidating disease etiology. This study employed MR to investigate the causal links between 731 immunophenotypes and PSC. The findings may open up new research avenues into PSC pathogenesis while expediting the search for early interventions and treatments.

## 2. Materials and methods

### 2.1. Data sources

**Figure 1.**
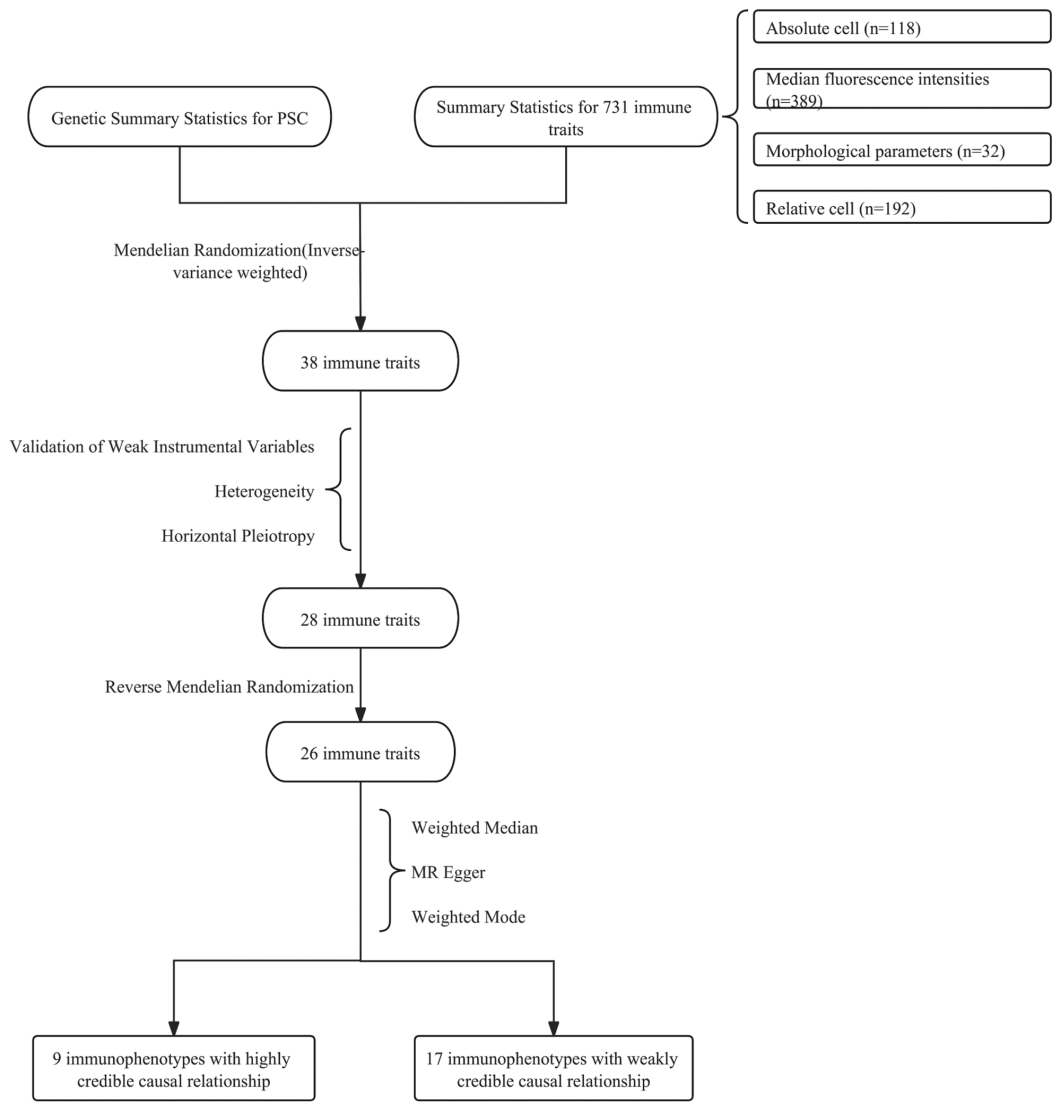
The complete pipeline of this study.

Figure 1 outlines the complete flowchart of this study.

The summary statistics for each immune trait used in this study were obtained from the GWAS Catalog (http://ftp.ebi.ac.uk/pub/databases/gwas/summary_statistics/, accession numbers GCST90001391 to GCST90002121). Furthermore, the original genome-wide association research on immunophenotypes included nonoverlapping data from 3757 European subjects.^[11]^ Of note, approximately 22 million single nucleotide polymorphisms (SNPs) genotyped with high-density arrays were imputed using a Sardinian sequence-based reference panel, and associations were assessed after adjusting for covariates.^[12]^ A total of 731 immunophenotypes were included in the study, including absolute cell (AC) counts (n = 118), median fluorescence intensities (MFI) indicating surface antigen levels (n = 389), morphological parameters (MPs) (n = 32), and relative cell (RC) counts (n = 192). The MFI, AC, and RC features contained B cells, CDCs, mature stages of T cells, monocytes, myeloid cells, TBNK (T cells, B cells, natural killer cells), and Treg panels, while the MP feature contained CDC and TBNK panels.

The genetic summary statistics for PSC were generated from GWAS, including 2871 PSC cases and 12,019 population controls.^[13]^ The summary statistics comprised 7891,602 SNPs from the MRC IEU OpenGWAS database (https://gwas.mrcieu.ac.uk/datasets/) (ID: ieu-a-1112).

This study was based on publicly available summary data and required no ethics approval or participant consent.

### 2.2. Selection of IVs

IVs for each immune trait with a *P*-value <1.0 × 10^−5^ were selected to improve the robustness of IV estimation. IVs were derived from independent loci by setting the linkage disequilibrium threshold at *R*^2^ < 0.001 and the clumping distance at 10,000 kb using the “TwoSampleMR” package on 1000 Genomes European data.

Relevant information for each SNP, including the effect allele, effect size including β-value, standard error, and *P*-value, was extracted from the MR results. Subsequently, we evaluated the proportion of variation explained (*R*^2^) and *F*-statistics to assess the strength of IVs. A threshold of *F* < 10 was used to define a “weak IV.” The “weak IV” will be excluded. The calculation formulas are as follows:


$$\begin{array}{l} R^{2}=2\times MAF\times \left(1-MAF\right)\times \beta^{2}; \end{array}$$

$$\begin{array}{l} F=R^{2}\left(n-k-1\right)/k\left(1-R^{2}\right). \end{array}$$

where “MAF” denotes the minor allele frequency of SNPs used as IVs, “*n*” denotes the sample size, and “*k*” denotes the number of IVs employed.^[14,15]^

### 2.3. Statistical analysis

Inverse-variance weighted (IVW) was employed as the primary analysis method. A *P*-value <0.05 denoted a causal relationship between the exposure and outcome variables. The IVW approach first leverages the Wald estimator to compute ratio estimations for individual SNPs and the delta method. It then aggregates the estimates generated from each SNP to obtain an overall causal effect estimate.^[16]^ IVW can provide the most precise effect estimations and is thus extensively employed in MR studies. To augment the robustness of the method, we used the weighted median, MR-Egger, and weighted mode methods to evaluate whether the observed association between immune traits and PSC was consistent with a causal effect.

Additionally, we assessed potential reverse associations between immune traits and PSC using reverse MR. Immune traits significantly associated with PSC in reverse analyses via IVW were excluded.

Cochran *Q*-statistics was employed to assess heterogeneity among the SNPs to establish the reliability and stability of the MR results in this study.^[17]^ A corresponding *P*-value of <0.05 denoted heterogeneity. Furthermore, the potential horizontal pleiotropy of the SNPs was evaluated using MR-Egger regression.^[18]^ A *P*-value of the intercept term <0.05 indicated potential horizontal pleiotropy of the SNPs. A “leave-one-out” analysis was performed to validate sensitivity and to investigate whether the causal association was driven by a single SNP. All MR analyses were performed using R version 4.2.3.

## 3. Results

Figure 1 outlines the complete pipeline of this study.

IVW analysis of the associations between 731 immunophenotypes and PSC risk yielded 38 immune traits with putative causal relationships. Subsequently, heterogeneity and horizontal pleiotropy were assessed to exclude traits with the likelihood of spurious causality. A final 28 immune traits were validated to have causal associations with PSC after stringent statistical filtration. We implemented reverse MR with the IVW method to assess the potential reverse effects of PSC on immunophenotypes to preclude reverse causation. Two immune traits exhibiting significant reverse associations were excluded from downstream analyses. *F*-statistics were computed for each SNP of the 26 immunophenotypes to evaluate the strength of IVs used in this study. The results revealed no “weak IVs” since all IVs had *F*-statistics surpassing 10 (Table S1, Supplemental Digital Content, http://links.lww.com/MD/M972).

**Figure 2.**
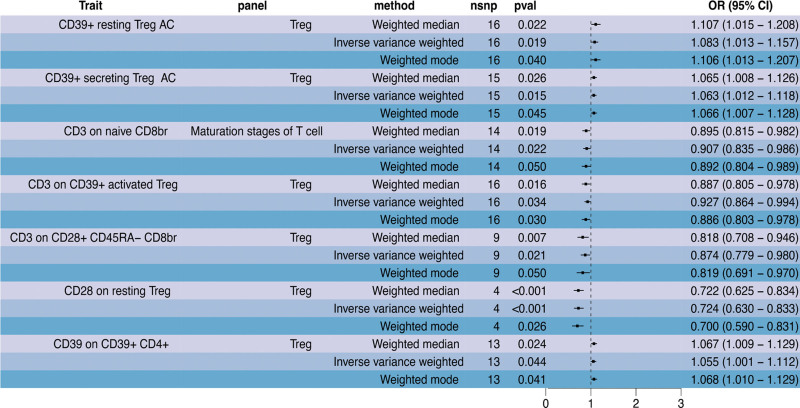
The forest plot of highly credible causal associations between 2 immune traits and primary sclerosing cholangitis risk by Mendelian Randomization analysis. CI = confidence interval, nSNP = number of single nucleotide polymorphism, OR = odds ratio.

The IVW analysis verified that the 26 immunophenotypes exhibited causal associations with PSC (Table S2, Supplemental Digital Content, http://links.lww.com/MD/M973). The heterogeneity test results (Table S3, Supplemental Digital Content, http://links.lww.com/MD/M974) and horizontal pleiotropy test results (Table S4, Supplemental Digital Content, http://links.lww.com/MD/M975) for the SNPs of these immunophenotypes are summarized in the supplementary data. The consistency of the observed immunophenotype-PSC associations was further evaluated using the weighted median, MR-Egger, and weighted mode methods to ensure the robustness of the findings. This sensitivity analysis revealed concordant associations for 2 immune traits across all 4 methods (Fig. 2). Specifically, the odds ratio (OR) of CD3 on HLA-DR+ CD4+ (TBNK panel) for PSC risk was 0.904 (95% confidence interval (CI): 0.828–0.986, *P = *.023) by IVW. The other 3 methods yielded similar effect sizes: MR-Egger OR 0.801 (95% CI: 0.659–0.974, *P = *.043); weighted median OR 0.853 (95% CI: 0.755–0.962, *P = *.010); and weighted mode OR 0.863 (95% CI: 0.765–0.975, *P = *.032). Additionally, the IVW OR of CD3 on secreting Treg (Treg panel) for PSC risk was 0.893 (95% CI: 0.823–0.969, *P = *.007). Comparable estimates were obtained using the 3 complementary methods: MR-Egger OR 0.843 (95% CI: 0.726–0.979, *P = *.047); weighted median OR 0.891 (95% CI: 0.811–0.980, *P = *.017); and weighted mode OR 0.895 (95% CI: 0.814–0.983, *P = *.039). These results demonstrate that both immunophenotypes had inverse associations with PSC risk (Figure S1, Supplemental Digital Content, http://links.lww.com/MD/M969).

**Figure 3.**
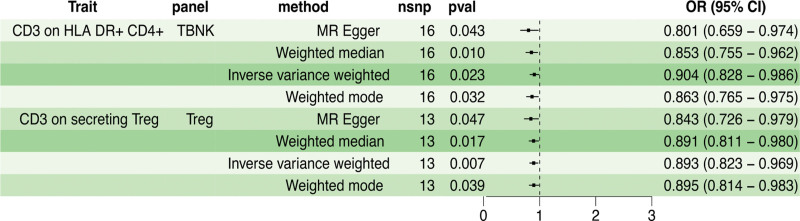
The forest plot of moderate credible causal associations between 7 immune traits and primary sclerosing cholangitis risk by Mendelian Randomization analysis. CI = confidence interval, nSNP =  number of single nucleotide polymorphism, OR = odds ratio.

IVW analysis initially revealed 7 immune traits potentially associated with PSC risk. However, in subsequent validation using weighted median, MR-Egger, and weighted mode methods, only weighted median and weighted mode corroborated the initial findings, whilst MR-Egger did not yield significant associations (Fig. 3).

The IVW analysis revealed that higher levels of CD39+ resting Treg AC (Treg panel) were associated with increased odds of PSC (OR = 1.083, 95% CI: 1.013–1.157, *P = *.019). The weighted median and weighted mode methods yielded similar effect size estimates. The elevated levels of CD39+ secreting Treg AC (Treg panel) were associated with a higher risk of PSC (IVW OR = 1.063, 95% CI: 1.012–1.118, *P = *.015), supporting the results from the other 2 methods. Furthermore, increased CD3 expression on naive CD8br (T cell maturation panel) was linked to reduced odds of PSC (IVW OR = 0.907, 95% CI: 0.835–0.986, *P* = .022), demonstrating consistency across the other 2 methods. Similarly, higher levels of CD3 on CD39+ activated Treg (Treg panel) were associated with a lower risk of PSC according to the IVW analysis (OR = 0.927, 95% CI: 0.864–0.994, *P = *.034), as well as the weighted median and weighted mode analyses. Moreover, increased levels of CD3 on CD28+ CD45RA− CD8br (Treg panel) were consistently associated with decreased odds of PSC based on the IVW analysis (OR = 0.874, 95% CI: 0.779–0.980, *P = *.021), weighted median, and weighted mode methods. Additionally, increased CD28 expression on resting Treg (Treg panel) significantly decreased the risk of PSC (IVW OR = 0.724, 95% CI: 0.630–0.833, *P = *5.95E-06), consistent with the findings from the other methods. Elevated levels of CD39 on CD39+ CD4+ (Treg panel) were associated with a higher risk of PSC (IVW OR = 1.055, 95% CI: 1.001–1.112, *P = *.044), consistently observed across all methods (Figure S2, Supplemental Digital Content, http://links.lww.com/MD/M970).

A “leave-one-out” sensitivity analysis was performed to investigate whether the causal associations between the aforementioned 9 immune traits and PSC were driven by any single SNP (Figure S3, Supplemental Digital Content, http://links.lww.com/MD/M971). The results demonstrated that the stepwise exclusion of each SNP did not substantially alter model effect estimates or qualitative inferences. Of note, both predictive outputs and interpretative conclusions were robust to the omission of any 1 SNP factor.

Furthermore, 17 additional immunophenotypes demonstrated potential causal links to PSC pathogenesis. However, 1 or none of the 3 validation methods verified these correlations. Larger samples may be necessary to definitively assess the proposed causal relationships between these 17 immune traits and PSC risk.

## 4. Discussion

This work explored the causal relationships between 731 immunophenotypes and PSC by leveraging extensive publicly available genetic data. This is the first MR analysis to investigate the causal effects of various immunophenotypes on PSC. We found evidence supporting the causal impact of PSC on 26 immunophenotypes across 4 types of immune traits (MFI, RC, AC, and MP) (*P < *.05), with 9 immunophenotypes demonstrating satisfactory credibility across validation analyses.

CD3 on HLA-DR+ CD4+ cells may constitute a subset of double-positive T lymphocytes expressing T- and B-cell markers. HLA-DR is an MHC class II cell surface receptor encoded by the human leukocyte antigen complex on chromosome 6 region 6P21.^[19]^ The present investigation provides the first evidence that CD3 on HLA-DR+ CD4+ can enhance the immune response of the body and may effectively clear dysregulated microbial molecules from the intestine entering through the portal circulation, thereby playing a protective role. However, its exact functional role remains to be elucidated. Similarly, studies of peripheral blood lymphocyte subsets in multiple sclerosis patients revealed that the proportion of HLA-DR+ CD4+ T cells was significantly lower than the controls.^[20]^

Regulatory T cells (Tregs) are critical for maintaining peripheral tolerance and exerting essential immunomodulatory functions in autoimmune diseases, allergies, tumor progression, transplant rejection, and infectious diseases.^[21,22]^ They can inhibit the expression of co-stimulatory molecules on antigen-presenting cells and the secretion of proinflammatory cytokines in an interleukin-10-dependent manner.^[23,24]^ An increased frequency of IL-10-secreting Treg cells was revealed to enhance resistance to autoimmune diseases in mice by increasing T helper 2 immune responses.^[25]^ Similarly, we found that CD3 secretion of Treg may protect against PSC pathogenesis. We hypothesize that CD3 secretion of Treg may protect biliary epithelial cells by releasing immunosuppressive factors that inhibit T cell activation and inflammatory cytokine production, alleviating immune-mediated biliary damage.

In addition, through MR analysis, we identified 7 immunophenotypes with moderately credible causal relationships with the risk of PSC. CD3 on naive CD8br is a subset of primary T lymphocytes recently emigrated from the thymus to peripheral lymphoid organs and are antigen inexperienced. These findings demonstrate that naive CD8+ T cells may contribute to the immunopathogenic mechanisms that underpin PSC. Augmentation of naive CD8+ T cell numbers and function could confer protection by suppressing the proliferation, activation, and effector capabilities of self-reactive T cells. The transcription factor BACH2 expressed in murine naive CD8+ T lymphocytes can restrain exaggerated immune responses and effector T cell activation.^[26]^ Furthermore, CD3 on CD39+ activated Treg constitutes an immunosuppressive subgroup of activated Treg cells, whereas CD28+ resting Treg is most likely an unstimulated Treg cell. In vivo, these cells can proliferate and migrate to inflammatory sites to exert immunosuppressive effects. The present study revealed that CD3 on CD39+ activated Treg and CD28+ resting Treg potentially regulate immune responses and attenuate T cell activation resulting from “gut lymphocyte homing,” contributing to PSC regulation. Similarly, circulating CD39+ Treg cells in patients play a role in regulating the course of multiple sclerosis.^[27]^ In this view, CD3 on CD28+ CD45RA− CD8br cells is likely a subset of memory/effector CD8+ T lymphocytes. These cells may engage in immune responses by cytotoxically clearing infected or aberrant cells. In this work, we anticipated that these cells may eliminate gut microbes and microbial molecules, preventing them from inducing aberrant biliary epithelial reactions via the portal vein, thereby ameliorating the development of PSC. CD39+ resting Treg AC, CD39+ secreting Treg AC, and CD39 on CD39+ CD4+ were identified as risk factors for PSC pathogenesis. However, CD39 can mediate immune suppression by degrading ATP and accumulating adenosine.^[28]^ Paradoxically, the present study revealed that these cells have a causal association with PSC. Therefore, we hypothesize that excessive accumulation of these Treg cells in hepatic tissues may conversely impair routine immune surveillance, allowing the persistence of pathogens and sustained activation of self-reactivity.

Moreover, the IVW technique identified 17 additional immunophenotypes that may be relevant to PSC pathogenesis, but 1 or none of the 3 validation methods verified these correlations. More data-driven investigations are required to validate these findings. In addition, further research is warranted to elucidate the intricate interplay between various immune cell subsets underlying the pathogenesis of this complex disease. Lastly, more comprehensive research is needed to unravel the precise mechanisms directing immune cell alterations in PSC.

This study performed a 2-sample MR analysis utilizing published genome-wide association study datasets. The conclusions are based on genetic IVs, demonstrating causal inference using multiple MR analytical approaches. The results are robust and unconfounded by horizontal pleiotropy or other variables. However, there are some limitations to this research. First, the lack of individual-level data precluded further stratified demographic analyses. Second, this study leveraged European databases; thus, the conclusions may not extend to other ethnicities, limiting the generalizability of our findings. Finally, we employed more relaxed thresholds when assessing the results, which may increase false positives while enabling a more comprehensive evaluation of the strong associations between immunophenotypes and PSC.

## 5. Conclusions

This study validated the causal associations of high credibility between 2 specific immunophenotypes and PSC, and of moderate credibility between 7 immunophenotypes and PSC using MR analysis. The results underscore the intricate interplay between the immune cells and PSC pathogenesis. Our study significantly reduced the influence of inherent confounding factors, reverse causation, and other biases. These findings may point researchers in new paths for deciphering the biological underpinnings of PSC, perhaps paving the way for further investigations into earlier intervention and treatment approaches.

## Author contributions

**Conceptualization:** Jie Zhou.

**Data curation:** Jie Zhou, Zhilin Liu.

**Formal analysis:** Jie Zhou.

**Writing—review & editing:** Jie Zhou.

**Supervision:** Haitao Wang, Yixin Xu.

**Investigation:** Yixin Xu.

## Supplementary Material














