# Supplementary material for: Dissecting the causal role of immunophenotypes in primary sclerosing cholangitis risk: A Mendelian randomization study
Source: Medicine (Baltimore). 2024 Jun 28;103(26):e38626. doi: 10.1097/MD.0000000000038626 (PMC11466166; doi:10.1097/MD.0000000000038626)
Supplement: Supplementary file 1 [file medi-103-e38626-s001.docx]

Table S1. F-statistics for each SNP of the 26 immunophenotypes

| Immunophenotypes | SNP | beta | SE | N | R2 | F |
| --- | --- | --- | --- | --- | --- | --- |
| Memory B cell AC |  |  |  |  |  |  |
|  | rs10018308 | 0.120 | 0.027 | 3656 | 0.005 | 19.971 |
|  | rs11653761 | -0.116 | 0.025 | 3656 | 0.006 | 21.939 |
|  | rs12763172 | 0.126 | 0.028 | 3656 | 0.006 | 20.454 |
|  | rs16867231 | 0.538 | 0.116 | 3656 | 0.006 | 21.610 |
|  | rs2879633 | -0.130 | 0.028 | 3656 | 0.006 | 21.968 |
|  | rs77643931 | 0.124 | 0.028 | 3656 | 0.005 | 19.767 |
|  | rs77762131 | 0.271 | 0.058 | 3656 | 0.006 | 21.906 |
| CD39+ resting Treg AC |  |  |  |  |  |  |
|  | rs11718018 | -0.114 | 0.025 | 3405 | 0.006 | 20.188 |
|  | rs117572834 | -1.021 | 0.214 | 3405 | 0.007 | 22.813 |
|  | rs117678654 | 0.602 | 0.121 | 3405 | 0.007 | 24.878 |
|  | rs12712610 | 0.191 | 0.027 | 3405 | 0.014 | 48.347 |
|  | rs138807420 | 1.024 | 0.204 | 3405 | 0.007 | 25.157 |
|  | rs138915779 | 1.188 | 0.228 | 3405 | 0.008 | 27.253 |
|  | rs143132810 | 0.636 | 0.079 | 3405 | 0.019 | 64.898 |
|  | rs17583875 | 2.134 | 0.223 | 3405 | 0.026 | 91.276 |
|  | rs185474383 | 0.529 | 0.087 | 3405 | 0.011 | 37.338 |
|  | rs190023756 | 0.614 | 0.121 | 3405 | 0.007 | 25.658 |
|  | rs2326054 | -0.225 | 0.050 | 3405 | 0.006 | 20.163 |
|  | rs35909109 | 0.360 | 0.061 | 3405 | 0.010 | 35.220 |
|  | rs4321083 | 0.137 | 0.031 | 3405 | 0.006 | 19.945 |
|  | rs4918971 | 0.807 | 0.021 | 3405 | 0.293 | 1408.652 |
|  | rs6488275 | -0.172 | 0.037 | 3405 | 0.006 | 21.062 |
|  | rs72809762 | 0.434 | 0.050 | 3405 | 0.022 | 76.654 |
|  | rs77520132 | 0.583 | 0.059 | 3405 | 0.028 | 97.550 |
| CD39+ resting Treg %resting Treg |  |  |  |  |  |  |
|  | rs115328872 | 1.259 | 0.199 | 3437 | 0.012 | 40.003 |
|  | rs11718018 | -0.118 | 0.025 | 3437 | 0.006 | 21.919 |
|  | rs117572834 | -0.965 | 0.214 | 3437 | 0.006 | 20.339 |
|  | rs12778837 | 0.201 | 0.027 | 3437 | 0.016 | 57.267 |
|  | rs141700904 | 0.531 | 0.113 | 3437 | 0.006 | 21.927 |
|  | rs1923692 | -0.572 | 0.053 | 3437 | 0.033 | 116.768 |
|  | rs2127464 | 0.285 | 0.063 | 3437 | 0.006 | 20.392 |
|  | rs2326054 | -0.223 | 0.050 | 3437 | 0.006 | 20.105 |
|  | rs4293064 | 0.243 | 0.043 | 3437 | 0.009 | 31.638 |
|  | rs4918962 | -0.888 | 0.020 | 3437 | 0.358 | 1915.321 |
|  | rs62416192 | 0.828 | 0.183 | 3437 | 0.006 | 20.443 |
|  | rs7077127 | -0.142 | 0.031 | 3437 | 0.006 | 21.211 |
|  | rs72809757 | 0.545 | 0.053 | 3437 | 0.030 | 107.700 |
|  | rs73196633 | -0.146 | 0.032 | 3437 | 0.006 | 21.355 |
|  | rs7328393 | -0.120 | 0.027 | 3437 | 0.006 | 20.015 |
|  | rs75834156 | -0.166 | 0.035 | 3437 | 0.007 | 23.033 |
|  | rs77718305 | 0.285 | 0.061 | 3437 | 0.006 | 21.636 |
|  | rs78809247 | -0.362 | 0.081 | 3437 | 0.006 | 19.872 |
|  | rs7963011 | 0.125 | 0.027 | 3437 | 0.006 | 21.045 |
| CD39+ resting Treg % CD4 Treg |  |  |  |  |  |  |
|  | rs113435341 | 2.267 | 0.217 | 3437 | 0.031 | 108.976 |
|  | rs117572834 | -1.108 | 0.214 | 3437 | 0.008 | 26.917 |
|  | rs11818051 | 0.700 | 0.072 | 3437 | 0.027 | 94.675 |
|  | rs12258674 | -0.151 | 0.034 | 3437 | 0.006 | 19.805 |
|  | rs12518588 | 0.124 | 0.025 | 3437 | 0.007 | 24.180 |
|  | rs12712610 | 0.201 | 0.027 | 3437 | 0.016 | 54.616 |
|  | rs12778837 | 0.197 | 0.027 | 3437 | 0.016 | 54.910 |
|  | rs1410600 | -0.222 | 0.025 | 3437 | 0.022 | 76.311 |
|  | rs150096082 | 14.690 | 2.928 | 3437 | 0.007 | 25.156 |
|  | rs17111341 | 0.685 | 0.050 | 3437 | 0.052 | 186.773 |
|  | rs182702572 | 0.546 | 0.074 | 3437 | 0.016 | 54.459 |
|  | rs2326054 | -0.231 | 0.050 | 3437 | 0.006 | 21.670 |
|  | rs35909109 | 0.381 | 0.060 | 3437 | 0.012 | 40.414 |
|  | rs4918969 | 0.843 | 0.021 | 3437 | 0.324 | 1643.233 |
|  | rs5755717 | 0.131 | 0.030 | 3437 | 0.006 | 19.598 |
|  | rs61821998 | 0.421 | 0.084 | 3437 | 0.007 | 24.991 |
|  | rs77520132 | 0.607 | 0.058 | 3437 | 0.031 | 108.642 |
|  | rs78809247 | -0.367 | 0.081 | 3437 | 0.006 | 20.429 |
|  | rs7963011 | 0.121 | 0.027 | 3437 | 0.006 | 19.731 |
|  | rs884599 | -0.111 | 0.025 | 3437 | 0.006 | 20.280 |
| CD39+ secreting Treg AC |  |  |  |  |  |  |
|  | rs10786199 | -0.193 | 0.029 | 3405 | 0.013 | 44.174 |
|  | rs10882655 | -0.959 | 0.020 | 3405 | 0.392 | 2197.521 |
|  | rs11598645 | -0.634 | 0.138 | 3405 | 0.006 | 21.044 |
|  | rs11655883 | 0.129 | 0.028 | 3405 | 0.006 | 21.437 |
|  | rs149951966 | 0.207 | 0.044 | 3405 | 0.006 | 22.219 |
|  | rs202211780 | 0.472 | 0.046 | 3405 | 0.030 | 104.365 |
|  | rs57516532 | 0.316 | 0.071 | 3405 | 0.006 | 19.873 |
|  | rs6005121 | -0.201 | 0.042 | 3405 | 0.007 | 23.077 |
|  | rs7267949 | -0.750 | 0.169 | 3405 | 0.006 | 19.714 |
|  | rs72809762 | 0.432 | 0.051 | 3405 | 0.020 | 71.049 |
|  | rs75270901 | 0.259 | 0.058 | 3405 | 0.006 | 19.804 |
|  | rs7570197 | 0.583 | 0.129 | 3405 | 0.006 | 20.480 |
|  | rs76464802 | -0.485 | 0.106 | 3405 | 0.006 | 21.099 |
|  | rs76710109 | -0.159 | 0.035 | 3405 | 0.006 | 20.801 |
|  | rs7937686 | 0.195 | 0.043 | 3405 | 0.006 | 20.038 |
| Basophil AC |  |  |  |  |  |  |
|  | rs11203701 | 0.157 | 0.034 | 1857 | 0.012 | 21.893 |
|  | rs138503959 | -0.480 | 0.108 | 1857 | 0.011 | 19.695 |
|  | rs2013837 | -0.159 | 0.033 | 1857 | 0.012 | 22.996 |
|  | rs35406957 | -0.164 | 0.036 | 1857 | 0.011 | 20.975 |
|  | rs3865444 | -0.486 | 0.039 | 1857 | 0.078 | 157.947 |
|  | rs3897098 | -0.156 | 0.035 | 1857 | 0.010 | 19.648 |
|  | rs6446560 | -0.147 | 0.033 | 1857 | 0.011 | 19.928 |
|  | rs74831462 | 2.771 | 0.576 | 1857 | 0.012 | 23.094 |
|  | rs78915471 | 3.266 | 0.707 | 1857 | 0.011 | 21.335 |
|  | rs9297816 | 0.179 | 0.035 | 1857 | 0.014 | 25.980 |
|  | rs9842941 | -0.197 | 0.038 | 1857 | 0.014 | 26.333 |
| DP (CD4+CD8+) %T cell |  |  |  |  |  |  |
|  | rs10490700 | 0.130 | 0.028 | 3668 | 0.006 | 21.055 |
|  | rs28459093 | -0.252 | 0.051 | 3668 | 0.006 | 23.958 |
|  | rs73095609 | -0.129 | 0.027 | 3668 | 0.006 | 22.400 |
|  | rs74197595 | 0.151 | 0.033 | 3668 | 0.006 | 20.362 |
| CD19 on IgD+ CD24- |  |  |  |  |  |  |
|  | rs11120838 | -0.276 | 0.061 | 3656 | 0.005 | 20.128 |
|  | rs112152495 | -0.574 | 0.129 | 3656 | 0.005 | 19.676 |
|  | rs112417310 | 0.524 | 0.089 | 3656 | 0.009 | 34.872 |
|  | rs113812519 | 0.531 | 0.091 | 3656 | 0.009 | 33.843 |
|  | rs12216684 | 0.264 | 0.053 | 3656 | 0.007 | 24.383 |
|  | rs1345010 | 0.192 | 0.041 | 3656 | 0.006 | 21.424 |
|  | rs1449311 | 0.127 | 0.027 | 3656 | 0.006 | 21.955 |
|  | rs148381451 | -0.270 | 0.056 | 3656 | 0.006 | 23.377 |
|  | rs17493333 | -0.349 | 0.077 | 3656 | 0.006 | 20.327 |
|  | rs1986507 | 0.140 | 0.031 | 3656 | 0.005 | 20.018 |
|  | rs2903728 | -0.184 | 0.039 | 3656 | 0.006 | 22.724 |
|  | rs34798965 | -0.200 | 0.045 | 3656 | 0.005 | 19.910 |
|  | rs75912463 | -3.140 | 0.677 | 3656 | 0.006 | 21.519 |
|  | rs764603 | 0.492 | 0.107 | 3656 | 0.006 | 21.140 |
| CD19 on IgD- CD24- |  |  |  |  |  |  |
|  | rs10192011 | 0.124 | 0.027 | 3655 | 0.006 | 20.775 |
|  | rs10906006 | 0.127 | 0.027 | 3655 | 0.006 | 21.618 |
|  | rs1164128 | 0.115 | 0.025 | 3655 | 0.006 | 20.818 |
|  | rs11643297 | 0.114 | 0.025 | 3655 | 0.005 | 20.038 |
|  | rs12968867 | -0.128 | 0.026 | 3655 | 0.006 | 23.525 |
|  | rs1453509 | -0.188 | 0.038 | 3655 | 0.007 | 24.450 |
|  | rs148597489 | -0.845 | 0.180 | 3655 | 0.006 | 22.021 |
|  | rs16884870 | 0.214 | 0.046 | 3655 | 0.006 | 21.737 |
|  | rs17315418 | 0.120 | 0.027 | 3655 | 0.005 | 20.069 |
|  | rs2613786 | -0.114 | 0.025 | 3655 | 0.006 | 20.267 |
|  | rs631691 | -0.111 | 0.025 | 3655 | 0.005 | 19.730 |
|  | rs73056862 | 0.271 | 0.058 | 3655 | 0.006 | 21.406 |
|  | rs73254441 | 0.395 | 0.089 | 3655 | 0.005 | 19.611 |
|  | rs7332415 | -0.233 | 0.050 | 3655 | 0.006 | 21.683 |
|  | rs75656751 | -0.351 | 0.079 | 3655 | 0.005 | 19.644 |
|  | rs75663256 | 0.190 | 0.043 | 3655 | 0.005 | 19.724 |
|  | rs76451648 | 0.278 | 0.058 | 3655 | 0.006 | 22.645 |
| CD25 on IgD+ CD38br |  |  |  |  |  |  |
|  | rs10047878 | -0.114 | 0.025 | 3656 | 0.006 | 20.809 |
|  | rs11616609 | -0.121 | 0.025 | 3656 | 0.006 | 22.551 |
|  | rs145198352 | -0.448 | 0.100 | 3656 | 0.006 | 20.249 |
|  | rs1828160 | -0.112 | 0.024 | 3656 | 0.006 | 21.820 |
|  | rs2566772 | 0.464 | 0.104 | 3656 | 0.005 | 19.890 |
|  | rs3793662 | -0.181 | 0.029 | 3656 | 0.011 | 39.117 |
|  | rs4129608 | -0.164 | 0.035 | 3656 | 0.006 | 22.412 |
|  | rs4721574 | 0.149 | 0.027 | 3656 | 0.008 | 29.268 |
|  | rs6092493 | -0.112 | 0.025 | 3656 | 0.006 | 20.321 |
|  | rs62626323 | 0.349 | 0.040 | 3656 | 0.020 | 76.313 |
|  | rs6550133 | -0.164 | 0.032 | 3656 | 0.007 | 26.485 |
| CD3 on naive CD8br |  |  |  |  |  |  |
|  | rs115280990 | 0.888 | 0.185 | 2910 | 0.008 | 23.124 |
|  | rs12994487 | -0.148 | 0.032 | 2910 | 0.007 | 21.065 |
|  | rs148965436 | -0.230 | 0.049 | 2910 | 0.007 | 21.545 |
|  | rs16940758 | -0.161 | 0.036 | 2910 | 0.007 | 20.515 |
|  | rs2413113 | 0.135 | 0.027 | 2910 | 0.008 | 23.993 |
|  | rs2714019 | -0.130 | 0.029 | 2910 | 0.007 | 20.134 |
|  | rs2995089 | 0.619 | 0.025 | 2910 | 0.170 | 595.343 |
|  | rs4396110 | -0.124 | 0.028 | 2910 | 0.007 | 19.893 |
|  | rs4657706 | 0.192 | 0.033 | 2910 | 0.011 | 33.316 |
|  | rs61814886 | 0.318 | 0.044 | 2910 | 0.017 | 51.576 |
|  | rs6479155 | -0.127 | 0.027 | 2910 | 0.007 | 21.412 |
|  | rs7416513 | 0.167 | 0.034 | 2910 | 0.008 | 24.686 |
|  | rs75624489 | -0.547 | 0.119 | 2910 | 0.007 | 20.978 |
|  | rs78132817 | -0.499 | 0.081 | 2910 | 0.013 | 37.538 |
| CD3 on HLA DR+ CD4+ |  |  |  |  |  |  |
|  | rs10933796 | 0.124 | 0.028 | 3060 | 0.006 | 19.623 |
|  | rs12596540 | 0.144 | 0.031 | 3060 | 0.007 | 21.906 |
|  | rs13072593 | 0.145 | 0.031 | 3060 | 0.007 | 21.408 |
|  | rs13170201 | -0.142 | 0.030 | 3060 | 0.007 | 21.811 |
|  | rs145799392 | -0.338 | 0.071 | 3060 | 0.007 | 22.701 |
|  | rs147209445 | 0.546 | 0.122 | 3060 | 0.006 | 19.914 |
|  | rs16867696 | -0.354 | 0.070 | 3060 | 0.008 | 25.704 |
|  | rs1723018 | 0.438 | 0.026 | 3060 | 0.086 | 287.433 |
|  | rs27265 | 0.194 | 0.037 | 3060 | 0.009 | 26.874 |
|  | rs2742060 | 0.175 | 0.036 | 3060 | 0.008 | 24.327 |
|  | rs4787901 | 0.129 | 0.028 | 3060 | 0.007 | 21.280 |
|  | rs55952666 | 0.254 | 0.056 | 3060 | 0.007 | 20.891 |
|  | rs59738549 | 0.387 | 0.061 | 3060 | 0.013 | 40.361 |
|  | rs6684311 | -0.205 | 0.039 | 3060 | 0.009 | 28.165 |
|  | rs6765892 | 0.172 | 0.037 | 3060 | 0.007 | 21.117 |
|  | rs75535842 | -0.279 | 0.057 | 3060 | 0.008 | 24.274 |
| CD3 on CD39+ resting Treg |  |  |  |  |  |  |
|  | rs12215509 | -0.124 | 0.028 | 2649 | 0.007 | 19.654 |
|  | rs12449842 | -0.281 | 0.063 | 2649 | 0.007 | 19.970 |
|  | rs143008344 | -0.348 | 0.076 | 2649 | 0.008 | 20.778 |
|  | rs150879948 | -1.461 | 0.329 | 2649 | 0.007 | 19.669 |
|  | rs16921089 | -0.734 | 0.162 | 2649 | 0.008 | 20.598 |
|  | rs1723016 | 0.327 | 0.028 | 2649 | 0.050 | 139.938 |
|  | rs2832129 | 0.158 | 0.034 | 2649 | 0.008 | 21.131 |
|  | rs61874707 | -1.009 | 0.211 | 2649 | 0.009 | 22.872 |
|  | rs704859 | 0.141 | 0.029 | 2649 | 0.009 | 23.589 |
|  | rs73001512 | -0.358 | 0.068 | 2649 | 0.010 | 28.002 |
|  | rs7741756 | -0.284 | 0.061 | 2649 | 0.008 | 21.906 |
|  | rs79844317 | -0.391 | 0.088 | 2649 | 0.007 | 19.831 |
| CD3 on CD39+ activated Treg |  |  |  |  |  |  |
|  | rs10491313 | 0.164 | 0.036 | 2919 | 0.007 | 20.792 |
|  | rs10882659 | -0.223 | 0.027 | 2919 | 0.023 | 67.275 |
|  | rs12064796 | 0.120 | 0.027 | 2919 | 0.007 | 19.747 |
|  | rs12138291 | -0.259 | 0.038 | 2919 | 0.016 | 45.988 |
|  | rs12401936 | 0.166 | 0.037 | 2919 | 0.007 | 19.631 |
|  | rs1389168 | -0.126 | 0.028 | 2919 | 0.007 | 20.674 |
|  | rs181383275 | 0.824 | 0.182 | 2919 | 0.007 | 20.559 |
|  | rs2949661 | 0.580 | 0.025 | 2919 | 0.154 | 530.081 |
|  | rs35055340 | -0.451 | 0.076 | 2919 | 0.012 | 35.076 |
|  | rs4685239 | -0.137 | 0.029 | 2919 | 0.008 | 22.275 |
|  | rs552999 | -0.176 | 0.030 | 2919 | 0.011 | 33.876 |
|  | rs55740714 | -0.304 | 0.063 | 2919 | 0.008 | 22.989 |
|  | rs55772918 | -0.211 | 0.048 | 2919 | 0.007 | 19.648 |
|  | rs59302791 | 0.375 | 0.080 | 2919 | 0.007 | 21.668 |
|  | rs6589911 | -0.228 | 0.049 | 2919 | 0.007 | 21.881 |
|  | rs79041458 | 1.286 | 0.278 | 2919 | 0.007 | 21.415 |
| CD3 on secreting Treg |  |  |  |  |  |  |
|  | rs115839712 | -0.433 | 0.097 | 2919 | 0.007 | 20.111 |
|  | rs116168222 | -0.444 | 0.098 | 2919 | 0.007 | 20.614 |
|  | rs142130759 | 0.689 | 0.136 | 2919 | 0.009 | 25.785 |
|  | rs142935756 | -0.941 | 0.198 | 2919 | 0.008 | 22.498 |
|  | rs2949661 | 0.615 | 0.025 | 2919 | 0.168 | 590.764 |
|  | rs4396110 | -0.131 | 0.028 | 2919 | 0.008 | 22.493 |
|  | rs4656440 | -0.355 | 0.063 | 2919 | 0.011 | 31.298 |
|  | rs57844740 | 0.375 | 0.081 | 2919 | 0.007 | 21.544 |
|  | rs6684311 | -0.266 | 0.039 | 2919 | 0.016 | 46.610 |
|  | rs7094641 | 0.139 | 0.031 | 2919 | 0.007 | 19.683 |
|  | rs75466245 | 0.264 | 0.059 | 2919 | 0.007 | 19.939 |
|  | rs78042544 | 0.427 | 0.093 | 2919 | 0.007 | 20.973 |
|  | rs8024202 | -0.136 | 0.028 | 2919 | 0.008 | 23.707 |
| CD3 on CD28+ CD45RA- CD8br |  |  |  |  |  |  |
|  | rs11219278 | -0.127 | 0.028 | 2919 | 0.007 | 20.922 |
|  | rs12132394 | 0.149 | 0.029 | 2919 | 0.009 | 27.088 |
|  | rs1723018 | 0.367 | 0.026 | 2919 | 0.062 | 194.379 |
|  | rs2949670 | 0.233 | 0.039 | 2919 | 0.012 | 34.794 |
|  | rs327018 | -0.207 | 0.046 | 2919 | 0.007 | 20.508 |
|  | rs6684311 | -0.239 | 0.038 | 2919 | 0.013 | 39.538 |
|  | rs78043002 | 0.325 | 0.069 | 2919 | 0.008 | 22.441 |
|  | rs8024202 | -0.121 | 0.027 | 2919 | 0.007 | 19.700 |
|  | rs8128515 | 0.158 | 0.033 | 2919 | 0.008 | 23.119 |
| CD3 on CD28+ CD45RA+ CD8br |  |  |  |  |  |  |
|  | rs117079896 | 0.779 | 0.163 | 2919 | 0.008 | 22.764 |
|  | rs12138291 | -0.271 | 0.038 | 2919 | 0.017 | 49.942 |
|  | rs12729972 | -0.125 | 0.028 | 2919 | 0.007 | 20.340 |
|  | rs12990220 | -0.175 | 0.036 | 2919 | 0.008 | 23.458 |
|  | rs1442353 | -0.131 | 0.029 | 2919 | 0.007 | 19.760 |
|  | rs2235394 | -0.131 | 0.027 | 2919 | 0.008 | 22.906 |
|  | rs2995089 | 0.602 | 0.025 | 2919 | 0.164 | 572.293 |
|  | rs4656440 | -0.338 | 0.063 | 2919 | 0.010 | 28.878 |
|  | rs6479155 | -0.137 | 0.027 | 2919 | 0.009 | 25.747 |
|  | rs73526900 | 0.450 | 0.096 | 2919 | 0.008 | 22.165 |
|  | rs7519927 | -0.190 | 0.028 | 2919 | 0.016 | 47.171 |
|  | rs78043002 | 0.348 | 0.070 | 2919 | 0.008 | 24.976 |
|  | rs79617518 | 0.620 | 0.129 | 2919 | 0.008 | 23.033 |
|  | rs79844317 | -0.387 | 0.087 | 2919 | 0.007 | 19.744 |
| CD3 on CD4 Treg |  |  |  |  |  |  |
|  | rs142394936 | 0.692 | 0.153 | 2919 | 0.007 | 20.433 |
|  | rs2595260 | -0.156 | 0.034 | 2919 | 0.007 | 20.751 |
|  | rs2949661 | 0.629 | 0.025 | 2919 | 0.180 | 639.749 |
|  | rs35055340 | -0.484 | 0.076 | 2919 | 0.014 | 40.294 |
|  | rs4396110 | -0.125 | 0.027 | 2919 | 0.007 | 21.082 |
|  | rs552999 | -0.153 | 0.030 | 2919 | 0.009 | 25.414 |
|  | rs57844740 | 0.382 | 0.080 | 2919 | 0.008 | 22.915 |
|  | rs6684311 | -0.276 | 0.038 | 2919 | 0.017 | 51.496 |
|  | rs6697436 | -0.203 | 0.042 | 2919 | 0.008 | 23.830 |
|  | rs73072986 | -0.275 | 0.062 | 2919 | 0.007 | 19.627 |
| CD28 on resting Treg |  |  |  |  |  |  |
|  | rs112900587 | -0.245 | 0.052 | 2919 | 0.008 | 22.424 |
|  | rs62184016 | -0.518 | 0.034 | 2919 | 0.076 | 238.730 |
|  | rs72923091 | 0.143 | 0.029 | 2919 | 0.008 | 23.694 |
|  | rs79223025 | 0.192 | 0.041 | 2919 | 0.007 | 21.808 |
| CD25 on CD45RA- CD4 not Treg |  |  |  |  |  |  |
|  | rs1000411 | 0.134 | 0.029 | 3435 | 0.006 | 20.921 |
|  | rs10509573 | -0.131 | 0.029 | 3435 | 0.006 | 20.044 |
|  | rs139194198 | 0.498 | 0.112 | 3435 | 0.006 | 19.662 |
|  | rs145627854 | 0.838 | 0.185 | 3435 | 0.006 | 20.571 |
|  | rs175717 | -0.133 | 0.028 | 3435 | 0.006 | 22.352 |
|  | rs181516190 | 0.500 | 0.086 | 3435 | 0.010 | 33.357 |
|  | rs35894198 | 0.181 | 0.038 | 3435 | 0.006 | 22.155 |
|  | rs4075635 | -0.116 | 0.026 | 3435 | 0.006 | 20.411 |
|  | rs4669451 | 0.140 | 0.029 | 3435 | 0.007 | 23.368 |
|  | rs61839660 | 0.611 | 0.055 | 3435 | 0.034 | 121.949 |
|  | rs9480540 | 0.813 | 0.183 | 3435 | 0.006 | 19.620 |
| CD25 on activated Treg |  |  |  |  |  |  |
|  | rs111871083 | 0.157 | 0.034 | 3435 | 0.006 | 21.009 |
|  | rs114746521 | -0.489 | 0.107 | 3435 | 0.006 | 20.839 |
|  | rs117690070 | 0.516 | 0.104 | 3435 | 0.007 | 24.793 |
|  | rs1332159 | -0.127 | 0.026 | 3435 | 0.007 | 24.368 |
|  | rs16903407 | 0.448 | 0.100 | 3435 | 0.006 | 20.090 |
|  | rs6939307 | -0.128 | 0.027 | 3435 | 0.007 | 22.880 |
|  | rs73215328 | -0.155 | 0.032 | 3435 | 0.007 | 23.626 |
|  | rs77205061 | 0.238 | 0.052 | 3435 | 0.006 | 20.795 |
|  | rs7999263 | 0.175 | 0.040 | 3435 | 0.006 | 19.617 |
| FSC-A on CD8br |  |  |  |  |  |  |
|  | rs10433403 | -0.268 | 0.060 | 3113 | 0.006 | 19.772 |
|  | rs10786450 | 0.449 | 0.096 | 3113 | 0.007 | 21.761 |
|  | rs115682796 | 0.466 | 0.103 | 3113 | 0.007 | 20.629 |
|  | rs143298995 | -0.136 | 0.027 | 3113 | 0.008 | 24.549 |
|  | rs1891421 | -0.189 | 0.040 | 3113 | 0.007 | 22.500 |
|  | rs240534 | 0.121 | 0.027 | 3113 | 0.006 | 19.942 |
|  | rs343066 | -0.114 | 0.023 | 3113 | 0.008 | 23.654 |
|  | rs75425029 | 0.136 | 0.030 | 3113 | 0.007 | 20.582 |
|  | rs7556141 | 0.426 | 0.096 | 3113 | 0.006 | 19.866 |
|  | rs855786 | -0.122 | 0.024 | 3113 | 0.008 | 26.636 |
| CCR2 on myeloid DC |  |  |  |  |  |  |
|  | rs116800975 | 0.301 | 0.066 | 2870 | 0.007 | 21.011 |
|  | rs11775437 | 1.197 | 0.260 | 2870 | 0.007 | 21.197 |
|  | rs139808988 | 0.706 | 0.155 | 2870 | 0.007 | 20.771 |
|  | rs16827093 | 0.210 | 0.045 | 2870 | 0.008 | 22.146 |
|  | rs2856092 | 0.141 | 0.030 | 2870 | 0.008 | 22.066 |
|  | rs35587265 | -0.422 | 0.059 | 2870 | 0.018 | 51.773 |
|  | rs4384829 | -0.125 | 0.028 | 2870 | 0.007 | 19.831 |
|  | rs969808 | -0.269 | 0.056 | 2870 | 0.008 | 22.932 |
| CD39 on CD39+ CD4+ |  |  |  |  |  |  |
|  | rs10882655 | -0.985 | 0.021 | 2920 | 0.427 | 2177.465 |
|  | rs10883000 | 0.150 | 0.028 | 2920 | 0.010 | 28.390 |
|  | rs11188782 | -0.213 | 0.038 | 2920 | 0.011 | 30.995 |
|  | rs11929716 | -0.328 | 0.068 | 2920 | 0.008 | 23.403 |
|  | rs12269710 | -0.473 | 0.095 | 2920 | 0.008 | 24.909 |
|  | rs12603021 | 0.208 | 0.044 | 2920 | 0.007 | 22.046 |
|  | rs1410600 | -0.233 | 0.028 | 2920 | 0.023 | 68.211 |
|  | rs147765250 | 0.346 | 0.077 | 2920 | 0.007 | 20.358 |
|  | rs148810393 | 0.319 | 0.068 | 2920 | 0.008 | 22.145 |
|  | rs28687532 | -0.131 | 0.028 | 2920 | 0.008 | 22.746 |
|  | rs2886870 | -0.125 | 0.028 | 2920 | 0.007 | 20.265 |
|  | rs2901833 | 0.270 | 0.028 | 2920 | 0.030 | 90.587 |
|  | rs79502391 | -0.181 | 0.040 | 2920 | 0.007 | 20.282 |
| CD80 on myeloid DC |  |  |  |  |  |  |
|  | rs11178308 | 0.875 | 0.194 | 2871 | 0.007 | 20.222 |
|  | rs1146465 | -0.137 | 0.028 | 2871 | 0.008 | 23.258 |
|  | rs12609290 | 0.392 | 0.084 | 2871 | 0.008 | 21.864 |
|  | rs12640679 | 0.134 | 0.029 | 2871 | 0.008 | 22.064 |
|  | rs2052324 | -0.183 | 0.037 | 2871 | 0.009 | 24.833 |
|  | rs2325259 | 0.200 | 0.028 | 2871 | 0.017 | 50.985 |
|  | rs329521 | 0.219 | 0.048 | 2871 | 0.007 | 20.411 |
|  | rs34687967 | 0.142 | 0.032 | 2871 | 0.007 | 19.939 |
|  | rs55752165 | -0.128 | 0.028 | 2871 | 0.007 | 20.390 |
|  | rs6787493 | 0.406 | 0.045 | 2871 | 0.027 | 80.570 |
|  | rs71639910 | 0.252 | 0.055 | 2871 | 0.007 | 21.299 |
|  | rs7757423 | -0.129 | 0.028 | 2871 | 0.007 | 21.043 |
|  | rs79015439 | -0.546 | 0.123 | 2871 | 0.007 | 19.719 |
|  | rs9650736 | 0.227 | 0.047 | 2871 | 0.008 | 23.301 |
| CD45 on CD33dim HLA DR+ CD11b- |  |  |  |  |  |  |
|  | rs12459419 | -0.223 | 0.044 | 1635 | 0.016 | 25.948 |
|  | rs1354185 | -0.237 | 0.051 | 1635 | 0.013 | 21.288 |
|  | rs1784661 | -0.167 | 0.038 | 1635 | 0.012 | 19.684 |
|  | rs2406420 | -0.177 | 0.038 | 1635 | 0.013 | 21.178 |
|  | rs2824282 | 0.167 | 0.036 | 1635 | 0.013 | 21.348 |
|  | rs376418616 | 0.701 | 0.150 | 1635 | 0.013 | 21.861 |
