# Supplementary material for: Dissecting the causal role of immunophenotypes in primary sclerosing cholangitis risk: A Mendelian randomization study
Source: Medicine (Baltimore). 2024 Jun 28;103(26):e38626. doi: 10.1097/MD.0000000000038626 (PMC11466166; doi:10.1097/MD.0000000000038626)
Supplement: Supplementary file 2 [file medi-103-e38626-s002.docx]

Table S2. Results of the Mendelian Randomization Analysis for the 26 immunophenotypes

| Traits (panel) | Method | nSNP | B | SE | *P-val* | OR | OR_lci95 | OR_uci95 |
| --- | --- | --- | --- | --- | --- | --- | --- | --- |
| Memory B cell AC (B cell) |  |  |  |  |  |  |  |  |
|  | MR Egger | 7 | -0.432 | 0.199 | 0.082 | 0.649 | 0.440 | 0.958 |
|  | Weighted median | 7 | -0.148 | 0.124 | 0.231 | 0.862 | 0.677 | 1.099 |
|  | Inverse variance weighted | 7 | -0.197 | 0.095 | 0.038 | 0.821 | 0.682 | 0.989 |
|  | Weighted mode | 7 | -0.170 | 0.149 | 0.299 | 0.844 | 0.630 | 1.131 |
| CD39+ resting Treg AC (Treg) |  |  |  |  |  |  |  |  |
|  | MR Egger | 16 | 0.065 | 0.043 | 0.158 | 1.067 | 0.980 | 1.161 |
|  | Weighted median | 16 | 0.102 | 0.044 | 0.022 | 1.107 | 1.015 | 1.208 |
|  | Inverse variance weighted | 16 | 0.080 | 0.034 | 0.019 | 1.083 | 1.013 | 1.157 |
|  | Weighted mode | 16 | 0.100 | 0.045 | 0.040 | 1.106 | 1.013 | 1.207 |
| CD39+ resting Treg %resting Treg (Treg) |  |  |  |  |  |  |  |  |
|  | MR Egger | 19 | 0.061 | 0.038 | 0.126 | 1.063 | 0.987 | 1.146 |
|  | Weighted median | 19 | 0.061 | 0.032 | 0.055 | 1.063 | 0.999 | 1.132 |
|  | Inverse variance weighted | 19 | 0.066 | 0.026 | 0.013 | 1.068 | 1.014 | 1.125 |
|  | Weighted mode | 19 | 0.064 | 0.032 | 0.063 | 1.067 | 1.001 | 1.137 |
| CD39+ resting Treg % CD4 Treg (Treg) |  |  |  |  |  |  |  |  |
|  | MR Egger | 20 | 0.018 | 0.011 | 0.140 | 1.018 | 0.995 | 1.041 |
|  | Weighted median | 20 | 0.015 | 0.013 | 0.254 | 1.015 | 0.990 | 1.041 |
|  | Inverse variance weighted | 20 | 0.022 | 0.011 | 0.045 | 1.022 | 1.001 | 1.044 |
|  | Weighted mode | 20 | 0.018 | 0.012 | 0.148 | 1.018 | 0.995 | 1.042 |
| CD39+ secreting Treg AC (Treg) |  |  |  |  |  |  |  |  |
|  | MR Egger | 15 | 0.060 | 0.042 | 0.176 | 1.061 | 0.978 | 1.152 |
|  | Weighted median | 15 | 0.063 | 0.028 | 0.026 | 1.065 | 1.008 | 1.126 |
|  | Inverse variance weighted | 15 | 0.062 | 0.025 | 0.015 | 1.063 | 1.012 | 1.118 |
|  | Weighted mode | 15 | 0.064 | 0.029 | 0.045 | 1.066 | 1.007 | 1.128 |
| Basophil AC (Myeloid cell) |  |  |  |  |  |  |  |  |
|  | MR Egger | 11 | 0.053 | 0.028 | 0.094 | 1.054 | 0.997 | 1.114 |
|  | Weighted median | 11 | 0.039 | 0.032 | 0.227 | 1.040 | 0.976 | 1.107 |
|  | Inverse variance weighted | 11 | 0.047 | 0.023 | 0.045 | 1.048 | 1.001 | 1.097 |
|  | Weighted mode | 11 | 0.047 | 0.026 | 0.098 | 1.048 | 0.996 | 1.103 |
| DP (CD4+CD8+) %T cell (TBNK) |  |  |  |  |  |  |  |  |
|  | MR Egger | 4 | -0.233 | 0.653 | 0.755 | 0.792 | 0.220 | 2.850 |
|  | Weighted median | 4 | 0.308 | 0.184 | 0.093 | 1.361 | 0.950 | 1.951 |
|  | Inverse variance weighted | 4 | 0.310 | 0.148 | 0.036 | 1.364 | 1.020 | 1.824 |
|  | Weighted mode | 4 | 0.440 | 0.235 | 0.158 | 1.552 | 0.979 | 2.461 |
| CD19 on IgD+ CD24- (B cell) |  |  |  |  |  |  |  |  |
|  | MR Egger | 14 | -0.112 | 0.053 | 0.057 | 0.894 | 0.806 | 0.992 |
|  | Weighted median | 14 | -0.099 | 0.060 | 0.099 | 0.905 | 0.805 | 1.019 |
|  | Inverse variance weighted | 14 | -0.089 | 0.042 | 0.036 | 0.915 | 0.843 | 0.994 |
|  | Weighted mode | 14 | -0.107 | 0.054 | 0.069 | 0.899 | 0.809 | 0.999 |
| CD19 on IgD- CD24- (B cell) |  |  |  |  |  |  |  |  |
|  | MR Egger | 17 | -0.016 | 0.106 | 0.880 | 0.984 | 0.799 | 1.211 |
|  | Weighted median | 17 | -0.142 | 0.081 | 0.081 | 0.868 | 0.740 | 1.018 |
|  | Inverse variance weighted | 17 | -0.123 | 0.054 | 0.024 | 0.885 | 0.795 | 0.984 |
|  | Weighted mode | 17 | -0.178 | 0.113 | 0.136 | 0.837 | 0.670 | 1.045 |
| CD25 on IgD+ CD38br (B cell) |  |  |  |  |  |  |  |  |
|  | MR Egger | 9 | -0.087 | 0.169 | 0.624 | 0.917 | 0.659 | 1.277 |
|  | Weighted median | 9 | 0.095 | 0.104 | 0.360 | 1.100 | 0.897 | 1.348 |
|  | Inverse variance weighted | 9 | 0.153 | 0.077 | 0.048 | 1.165 | 1.001 | 1.355 |
|  | Weighted mode | 9 | 0.055 | 0.129 | 0.680 | 1.057 | 0.820 | 1.362 |
| CD3 on naive CD8br (Maturation stages of T cell) |  |  |  |  |  |  |  |  |
|  | MR Egger | 14 | -0.127 | 0.071 | 0.100 | 0.881 | 0.766 | 1.013 |
|  | Weighted median | 14 | -0.111 | 0.048 | 0.019 | 0.895 | 0.815 | 0.982 |
|  | Inverse variance weighted | 14 | -0.097 | 0.042 | 0.022 | 0.907 | 0.835 | 0.986 |
|  | Weighted mode | 14 | -0.115 | 0.053 | 0.050 | 0.892 | 0.804 | 0.989 |
| CD3 on HLA DR+ CD4+ (TBNK) |  |  |  |  |  |  |  |  |
|  | MR Egger | 16 | -0.222 | 0.100 | 0.043 | 0.801 | 0.659 | 0.974 |
|  | Weighted median | 16 | -0.159 | 0.062 | 0.010 | 0.853 | 0.755 | 0.962 |
|  | Inverse variance weighted | 16 | -0.101 | 0.045 | 0.023 | 0.904 | 0.828 | 0.986 |
|  | Weighted mode | 16 | -0.147 | 0.062 | 0.032 | 0.863 | 0.765 | 0.975 |
| CD3 on CD39+ resting Treg (Treg) |  |  |  |  |  |  |  |  |
|  | MR Egger | 12 | -0.040 | 0.057 | 0.501 | 0.961 | 0.860 | 1.074 |
|  | Weighted median | 12 | -0.067 | 0.058 | 0.251 | 0.935 | 0.834 | 1.048 |
|  | Inverse variance weighted | 12 | -0.087 | 0.038 | 0.024 | 0.917 | 0.850 | 0.988 |
|  | Weighted mode | 12 | -0.054 | 0.061 | 0.391 | 0.947 | 0.841 | 1.067 |
| CD3 on CD39+ activated Treg (Treg) |  |  |  |  |  |  |  |  |
|  | MR Egger | 16 | -0.050 | 0.062 | 0.430 | 0.951 | 0.842 | 1.074 |
|  | Weighted median | 16 | -0.119 | 0.050 | 0.016 | 0.887 | 0.805 | 0.978 |
|  | Inverse variance weighted | 16 | -0.076 | 0.036 | 0.034 | 0.927 | 0.864 | 0.994 |
|  | Weighted mode | 16 | -0.121 | 0.050 | 0.030 | 0.886 | 0.803 | 0.978 |
| CD3 on secreting Treg (Treg) |  |  |  |  |  |  |  |  |
|  | MR Egger | 13 | -0.171 | 0.076 | 0.047 | 0.843 | 0.726 | 0.979 |
|  | Weighted median | 13 | -0.115 | 0.048 | 0.017 | 0.891 | 0.811 | 0.980 |
|  | Inverse variance weighted | 13 | -0.113 | 0.042 | 0.007 | 0.893 | 0.823 | 0.969 |
|  | Weighted mode | 13 | -0.111 | 0.048 | 0.039 | 0.895 | 0.814 | 0.983 |
| CD3 on CD28+ CD45RA- CD8br (Treg) |  |  |  |  |  |  |  |  |
|  | MR Egger | 9 | -0.170 | 0.149 | 0.290 | 0.844 | 0.631 | 1.129 |
|  | Weighted median | 9 | -0.201 | 0.074 | 0.007 | 0.818 | 0.708 | 0.946 |
|  | Inverse variance weighted | 9 | -0.135 | 0.058 | 0.021 | 0.874 | 0.779 | 0.980 |
|  | Weighted mode | 9 | -0.200 | 0.087 | 0.050 | 0.819 | 0.691 | 0.970 |
| CD3 on CD28+ CD45RA+ CD8br (Treg) |  |  |  |  |  |  |  |  |
|  | MR Egger | 14 | -0.097 | 0.065 | 0.166 | 0.908 | 0.799 | 1.032 |
|  | Weighted median | 14 | -0.103 | 0.048 | 0.032 | 0.902 | 0.821 | 0.991 |
|  | Inverse variance weighted | 14 | -0.087 | 0.038 | 0.020 | 0.916 | 0.851 | 0.986 |
|  | Weighted mode | 14 | -0.111 | 0.054 | 0.060 | 0.895 | 0.806 | 0.994 |
| CD3 on CD4 Treg (Treg) |  |  |  |  |  |  |  |  |
|  | MR Egger | 10 | -0.141 | 0.073 | 0.091 | 0.868 | 0.752 | 1.003 |
|  | Weighted median | 10 | -0.108 | 0.045 | 0.015 | 0.897 | 0.822 | 0.979 |
|  | Inverse variance weighted | 10 | -0.091 | 0.041 | 0.025 | 0.913 | 0.843 | 0.989 |
|  | Weighted mode | 10 | -0.095 | 0.051 | 0.094 | 0.909 | 0.823 | 1.005 |
| CD28 on resting Treg (Treg) |  |  |  |  |  |  |  |  |
|  | MR Egger | 4 | -0.472 | 0.145 | 0.083 | 0.624 | 0.469 | 0.829 |
|  | Weighted median | 4 | -0.326 | 0.073 | 0.000 | 0.722 | 0.625 | 0.834 |
|  | Inverse variance weighted | 4 | -0.322 | 0.071 | 0.000 | 0.724 | 0.630 | 0.833 |
|  | Weighted mode | 4 | -0.357 | 0.087 | 0.026 | 0.700 | 0.590 | 0.831 |
| CD25 on CD45RA- CD4 not Treg (Treg) |  |  |  |  |  |  |  |  |
|  | MR Egger | 11 | -0.165 | 0.093 | 0.111 | 0.848 | 0.706 | 1.018 |
|  | Weighted median | 11 | -0.077 | 0.074 | 0.297 | 0.926 | 0.801 | 1.070 |
|  | Inverse variance weighted | 11 | -0.124 | 0.061 | 0.044 | 0.884 | 0.784 | 0.997 |
|  | Weighted mode | 11 | -0.063 | 0.083 | 0.463 | 0.939 | 0.798 | 1.104 |
| CD25 on activated Treg (Treg) |  |  |  |  |  |  |  |  |
|  | MR Egger | 9 | -0.044 | 0.196 | 0.829 | 0.957 | 0.652 | 1.406 |
|  | Weighted median | 9 | -0.127 | 0.118 | 0.281 | 0.881 | 0.699 | 1.110 |
|  | Inverse variance weighted | 9 | -0.209 | 0.090 | 0.021 | 0.812 | 0.680 | 0.969 |
|  | Weighted mode | 9 | -0.105 | 0.163 | 0.536 | 0.900 | 0.654 | 1.238 |
| FSC-A on CD8br (TBNK) |  |  |  |  |  |  |  |  |
|  | MR Egger | 10 | 0.135 | 0.194 | 0.508 | 1.144 | 0.782 | 1.674 |
|  | Weighted median | 10 | 0.192 | 0.118 | 0.104 | 1.212 | 0.961 | 1.527 |
|  | Inverse variance weighted | 10 | 0.202 | 0.088 | 0.021 | 1.224 | 1.031 | 1.454 |
|  | Weighted mode | 10 | 0.213 | 0.161 | 0.218 | 1.237 | 0.903 | 1.696 |
| CCR2 on myeloid DC (cDC) |  |  |  |  |  |  |  |  |
|  | MR Egger | 8 | -0.151 | 0.067 | 0.067 | 0.860 | 0.754 | 0.982 |
|  | Weighted median | 8 | -0.095 | 0.065 | 0.144 | 0.910 | 0.801 | 1.033 |
|  | Inverse variance weighted | 8 | -0.116 | 0.048 | 0.015 | 0.890 | 0.810 | 0.978 |
|  | Weighted mode | 8 | -0.102 | 0.058 | 0.125 | 0.903 | 0.806 | 1.013 |
| CD39 on CD39+ CD4+ (Treg) |  |  |  |  |  |  |  |  |
|  | MR Egger | 13 | 0.082 | 0.041 | 0.069 | 1.085 | 1.002 | 1.175 |
|  | Weighted median | 13 | 0.065 | 0.029 | 0.024 | 1.067 | 1.009 | 1.129 |
|  | Inverse variance weighted | 13 | 0.054 | 0.027 | 0.044 | 1.055 | 1.001 | 1.112 |
|  | Weighted mode | 13 | 0.065 | 0.029 | 0.041 | 1.068 | 1.010 | 1.129 |
| CD80 on myeloid DC (cDC) |  |  |  |  |  |  |  |  |
|  | MR Egger | 14 | 0.020 | 0.156 | 0.898 | 1.020 | 0.752 | 1.384 |
|  | Weighted median | 14 | 0.096 | 0.085 | 0.260 | 1.100 | 0.932 | 1.300 |
|  | Inverse variance weighted | 14 | 0.136 | 0.065 | 0.035 | 1.146 | 1.009 | 1.301 |
|  | Weighted mode | 14 | 0.043 | 0.111 | 0.705 | 1.044 | 0.840 | 1.297 |
| CD45 on CD33dim HLA DR+ CD11b- (Myeloid cell) |  |  |  |  |  |  |  |  |
|  | MR Egger | 6 | -0.148 | 0.402 | 0.731 | 0.862 | 0.392 | 1.896 |
|  | Weighted median | 6 | -0.228 | 0.106 | 0.031 | 0.796 | 0.646 | 0.980 |
|  | Inverse variance weighted | 6 | -0.170 | 0.081 | 0.036 | 0.843 | 0.719 | 0.989 |
|  | Weighted mode | 6 | -0.266 | 0.157 | 0.150 | 0.766 | 0.564 | 1.042 |
|  | Method | nSNP | B | SE | *P-val* | OR | OR_lci95 | OR_uci95 |
