# Supplementary material for: Dissecting the causal role of immunophenotypes in primary sclerosing cholangitis risk: A Mendelian randomization study
Source: Medicine (Baltimore). 2024 Jun 28;103(26):e38626. doi: 10.1097/MD.0000000000038626 (PMC11466166; doi:10.1097/MD.0000000000038626)
Supplement: Supplementary file 3 [file medi-103-e38626-s003.docx]

Table S3. Corroboration of heterogeneity examination among single nucleotide polymorphisms

| Traits (panel) | Method | Q | Q_df | *Q_pval* |
| --- | --- | --- | --- | --- |
| Memory B cell AC (B cell) |  |  |  |  |
|  | MR Egger | 1.000 | 5 | 0.963 |
|  | Inverse variance weighted | 2.812 | 6 | 0.832 |
| CD39+ resting Treg AC (Treg) |  |  |  |  |
|  | MR Egger | 10.612 | 14 | 0.716 |
|  | Inverse variance weighted | 10.917 | 15 | 0.758 |
| CD39+ resting Treg %resting Treg (Treg) |  |  |  |  |
|  | MR Egger | 10.733 | 17 | 0.870 |
|  | Inverse variance weighted | 10.764 | 18 | 0.904 |
| CD39+ resting Treg % CD4 Treg (Treg) |  |  |  |  |
|  | MR Egger | 12.029 | 18 | 0.846 |
|  | Inverse variance weighted | 12.980 | 19 | 0.840 |
| CD39+ secreting Treg AC (Treg) |  |  |  |  |
|  | MR Egger | 9.568 | 13 | 0.729 |
|  | Inverse variance weighted | 9.572 | 14 | 0.793 |
| Basophil AC (Myeloid cell) |  |  |  |  |
|  | MR Egger | 9.368 | 9 | 0.404 |
|  | Inverse variance weighted | 9.527 | 10 | 0.483 |
| DP (CD4+CD8+) %T cell (TBNK) |  |  |  |  |
|  | MR Egger | 0.400 | 2 | 0.819 |
|  | Inverse variance weighted | 1.129 | 3 | 0.770 |
| CD19 on IgD+ CD24- (B cell) |  |  |  |  |
|  | MR Egger | 6.209 | 12 | 0.905 |
|  | Inverse variance weighted | 6.723 | 13 | 0.916 |
| CD19 on IgD- CD24- (B cell) |  |  |  |  |
|  | MR Egger | 11.475 | 15 | 0.718 |
|  | Inverse variance weighted | 12.839 | 16 | 0.684 |
| CD25 on IgD+ CD38br (B cell) |  |  |  |  |
|  | MR Egger | 5.300 | 7 | 0.623 |
|  | Inverse variance weighted | 7.839 | 8 | 0.449 |
| CD3 on naive CD8br (Maturation stages of T cell) |  |  |  |  |
|  | MR Egger | 16.694 | 12 | 0.161 |
|  | Inverse variance weighted | 17.077 | 13 | 0.196 |
| CD3 on HLA DR+ CD4+ (TBNK) |  |  |  |  |
|  | MR Egger | 9.346 | 14 | 0.808 |
|  | Inverse variance weighted | 11.162 | 15 | 0.741 |
| CD3 on CD39+ resting Treg (Treg) |  |  |  |  |
|  | MR Egger | 8.784 | 10 | 0.553 |
|  | Inverse variance weighted | 10.053 | 11 | 0.526 |
| CD3 on CD39+ activated Treg (Treg) |  |  |  |  |
|  | MR Egger | 5.440 | 14 | 0.979 |
|  | Inverse variance weighted | 5.694 | 15 | 0.984 |
| CD3 on secreting Treg (Treg) |  |  |  |  |
|  | MR Egger | 11.903 | 11 | 0.371 |
|  | Inverse variance weighted | 12.783 | 12 | 0.385 |
| CD3 on CD28+ CD45RA- CD8br (Treg) |  |  |  |  |
|  | MR Egger | 6.923 | 7 | 0.437 |
|  | Inverse variance weighted | 6.989 | 8 | 0.538 |
| CD3 on CD28+ CD45RA+ CD8br (Treg) |  |  |  |  |
|  | MR Egger | 6.436 | 12 | 0.893 |
|  | Inverse variance weighted | 6.464 | 13 | 0.928 |
| CD3 on CD4 Treg (Treg) |  |  |  |  |
|  | MR Egger | 7.981 | 8 | 0.435 |
|  | Inverse variance weighted | 8.653 | 9 | 0.470 |
| CD28 on resting Treg (Treg) |  |  |  |  |
|  | MR Egger | 0.678 | 2 | 0.712 |
|  | Inverse variance weighted | 2.083 | 3 | 0.555 |
| CD25 on CD45RA- CD4 not Treg (Treg) |  |  |  |  |
|  | MR Egger | 16.617 | 9 | 0.055 |
|  | Inverse variance weighted | 17.293 | 10 | 0.068 |
| CD25 on activated Treg (Treg) |  |  |  |  |
|  | MR Egger | 1.736 | 7 | 0.973 |
|  | Inverse variance weighted | 2.632 | 8 | 0.955 |
| FSC-A on CD8br (TBNK) |  |  |  |  |
|  | MR Egger | 6.344 | 8 | 0.609 |
|  | Inverse variance weighted | 6.496 | 9 | 0.689 |
| CCR2 on myeloid DC (cDC) |  |  |  |  |
|  | MR Egger | 5.987 | 6 | 0.425 |
|  | Inverse variance weighted | 6.506 | 7 | 0.482 |
| CD39 on CD39+ CD4+ (Treg) |  |  |  |  |
|  | MR Egger | 11.900 | 11 | 0.371 |
|  | Inverse variance weighted | 12.818 | 12 | 0.382 |
| CD80 on myeloid DC (cDC) |  |  |  |  |
|  | MR Egger | 14.919 | 12 | 0.246 |
|  | Inverse variance weighted | 15.759 | 13 | 0.262 |
| CD45 on CD33dim HLA DR+ CD11b- (Myeloid cell) |  |  |  |  |
|  | MR Egger | 3.874 | 4 | 0.423 |
|  | Inverse variance weighted | 3.877 | 5 | 0.567 |
