# Supplementary material for: Dissecting the causal role of immunophenotypes in primary sclerosing cholangitis risk: A Mendelian randomization study
Source: Medicine (Baltimore). 2024 Jun 28;103(26):e38626. doi: 10.1097/MD.0000000000038626 (PMC11466166; doi:10.1097/MD.0000000000038626)
Supplement: Supplementary file 4 [file medi-103-e38626-s004.docx]

| Traits (panel) | egger_intercept | se | p-val |
| --- | --- | --- | --- |
| Memory B cell AC (B cell) | 0.042 | 0.031 | 0.236 |
| CD39+ resting Treg AC (Treg) | 0.011 | 0.020 | 0.589 |
| CD39+ resting Treg %resting Treg (Treg) | 0.003 | 0.016 | 0.863 |
| CD39+ resting Treg % CD4 Treg (Treg) | 0.011 | 0.011 | 0.342 |
| CD39+ secreting Treg AC (Treg) | 0.001 | 0.020 | 0.954 |
| Basophil AC (Myeloid cell) | -0.006 | 0.015 | 0.705 |
| DP (CD4+CD8+) %T cell (TBNK) | 0.082 | 0.097 | 0.483 |
| CD19 on IgD+ CD24- (B cell) | 0.014 | 0.020 | 0.487 |
| CD19 on IgD- CD24- (B cell) | -0.023 | 0.020 | 0.261 |
| CD25 on IgD+ CD38br (B cell) | 0.052 | 0.032 | 0.155 |
| CD3 on naive CD8br (Maturation stages of T cell) | 0.011 | 0.020 | 0.609 |
| CD3 on HLA DR+ CD4+ (TBNK) | 0.031 | 0.023 | 0.199 |
| CD3 on CD39+ resting Treg (Treg) | -0.025 | 0.022 | 0.286 |
| CD3 on CD39+ activated Treg (Treg) | -0.010 | 0.020 | 0.622 |
| CD3 on secreting Treg (Treg) | 0.024 | 0.026 | 0.387 |
| CD3 on CD28+ CD45RA- CD8br (Treg) | 0.008 | 0.033 | 0.804 |
| CD3 on CD28+ CD45RA+ CD8br (Treg) | 0.003 | 0.020 | 0.868 |
| CD3 on CD4 Treg (Treg) | 0.021 | 0.025 | 0.436 |
| CD28 on resting Treg (Treg) | 0.061 | 0.051 | 0.358 |
| CD25 on CD45RA- CD4 not Treg (Treg) | 0.017 | 0.028 | 0.560 |
| CD25 on activated Treg (Treg) | -0.035 | 0.037 | 0.375 |
| FSC-A on CD8br (TBNK) | 0.013 | 0.034 | 0.707 |
| CCR2 on myeloid DC (cDC) | 0.019 | 0.026 | 0.498 |
| CD39 on CD39+ CD4+ (Treg) | -0.016 | 0.017 | 0.377 |
| CD80 on myeloid DC (cDC) | 0.025 | 0.030 | 0.427 |
| CD45 on CD33dim HLA DR+ CD11b- (Myeloid cell) | -0.004 | 0.079 | 0.958 |

Table S4. Validation of horizontal pleiotropy among the single nucleotide polymorphisms
