# Supplementary figures and images for: Dissecting the causal role of immunophenotypes in primary sclerosing cholangitis risk: A Mendelian randomization study
Source: Medicine (Baltimore). 2024 Jun 28;103(26):e38626. doi: 10.1097/MD.0000000000038626 (PMC11466166; doi:10.1097/MD.0000000000038626)

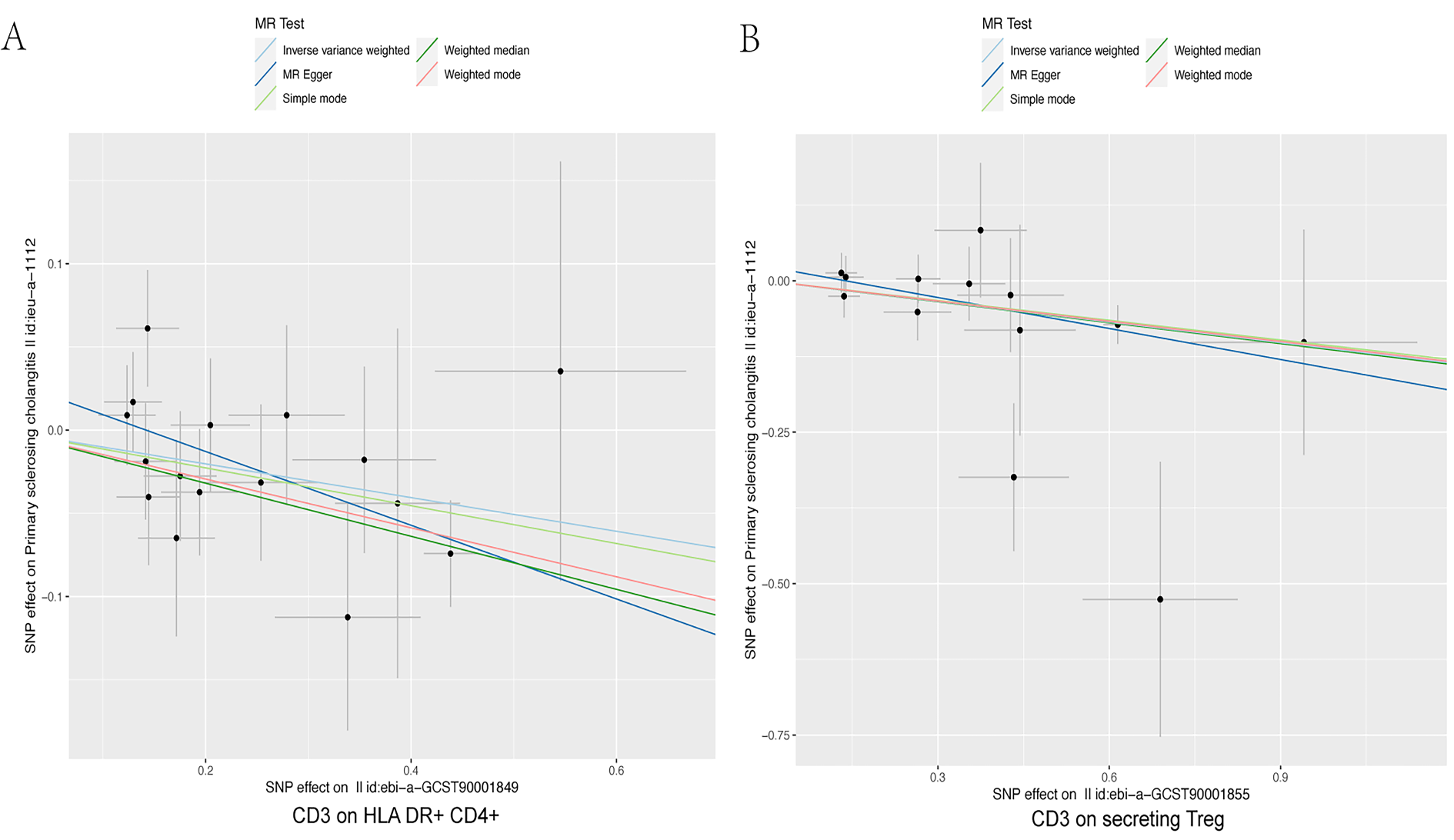

Supplement: Supplementary file 5 [file medi-103-e38626-s005.tif]

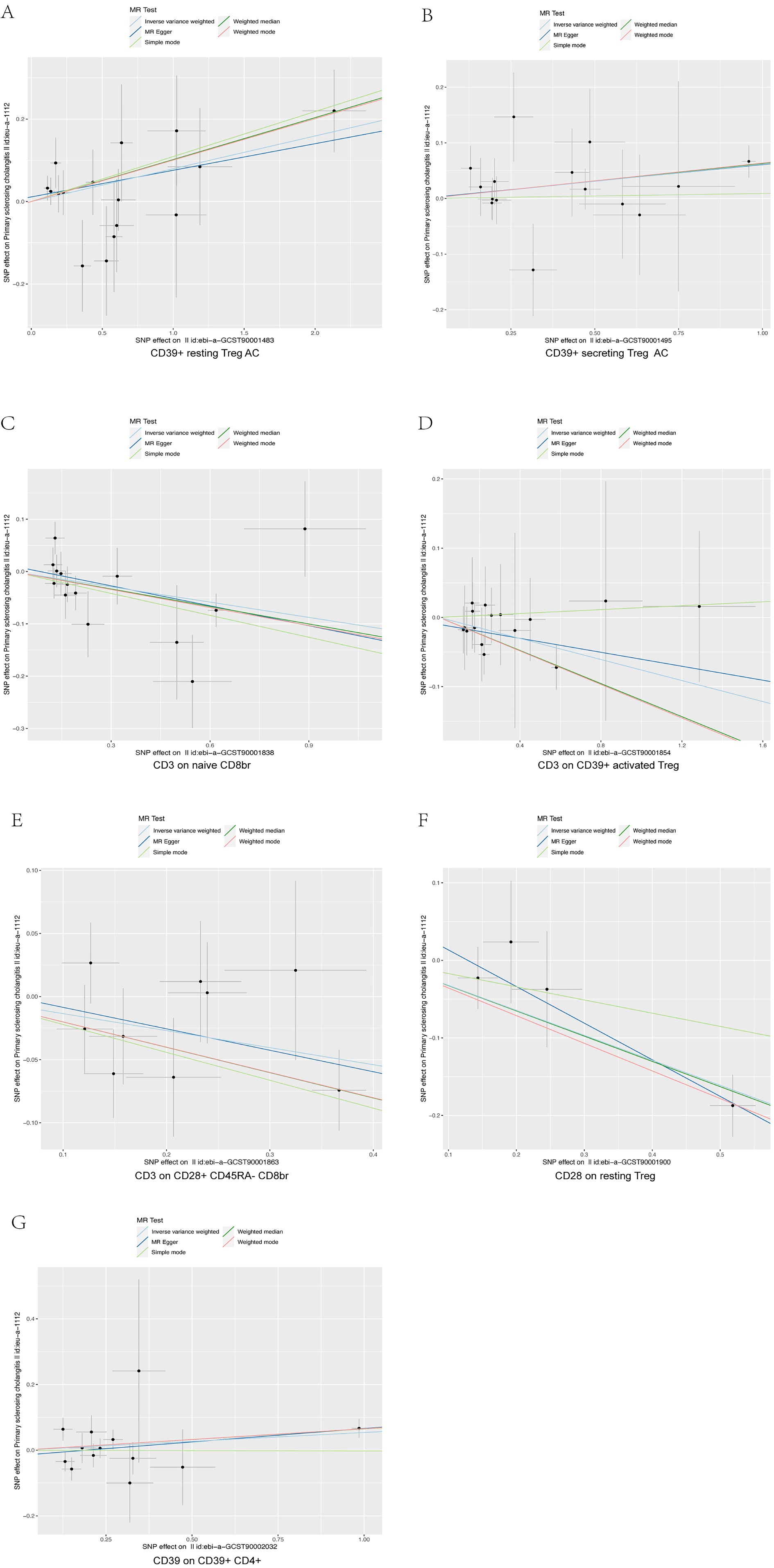

Supplement: Supplementary file 6 [file medi-103-e38626-s006.jpeg]

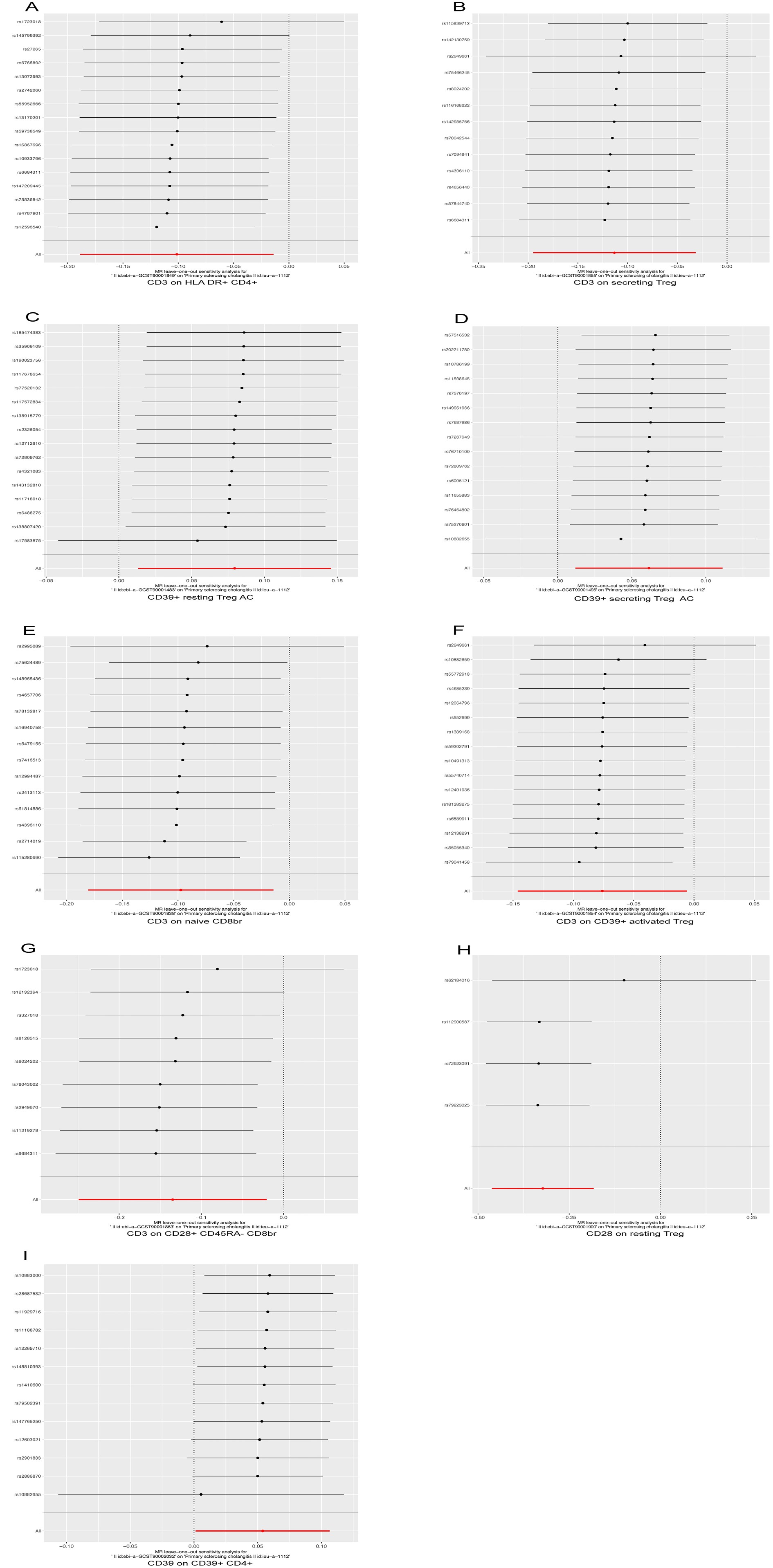

Supplement: Supplementary file 7 [file medi-103-e38626-s007.jpeg]
